# Global Environmental Change: What Can Health Care Providers and the Environmental Health Community Do About It Now?

**DOI:** 10.1289/ehp.9313

**Published:** 2006-09-05

**Authors:** Brian S. Schwartz, Cindy Parker, Thomas A. Glass, Howard Hu

**Affiliations:** 1 Department of Environmental Health Sciences and; 2 Department of Epidemiology, Johns Hopkins Bloomberg School of Public Health, Baltimore, Maryland, USA; 3 Department of Medicine, Johns Hopkins School of Medicine, Baltimore, Maryland, USA; 4 Department of Environmental Health Sciences, University of Michigan School of Public Health, Ann Arbor, Michigan, USA

**Keywords:** behavior change, climate change, health impacts

## Abstract

The debate about whether global environmental change is real is now over; in its wake is the realization that it is happening more rapidly than predicted. These changes constitute a profound challenge to human health, both as a direct threat and as a promoter of other risks. We call on health care providers to inform themselves about these issues and to become agents of change in their communities. It is our responsibility as clinicians to educate patients and their communities on the connections between regressive policies, unsustainable behaviors, global environmental changes, and threats to health and security. We call on professional organizations to assist in educating their members about these issues, in helping clinicians practice behavior change with their patients, and in adding their voices to this issue in our statehouses and Congress. We call for the development of carbon- and other environmental-labeling of consumer products so individuals can make informed choices; we also call for the rapid implementation of policies that provide tangible economic incentives for choosing environmentally sustainable products and services. We urge the environmental health community to take up the challenge of developing a global environmental health index that will incorporate human health into available “planetary health” metrics and that can be used as a policy tool to evaluate the impact of interventions and document spatial and temporal shifts in the healthfulness of local areas. Finally, we urge our political, business, public health, and academic leaders to heed these environmental warnings and quickly develop regulatory and policy solutions so that the health of populations and the integrity of their environments will be ensured for future generations.

On its 3 April 2006 cover *Time* magazine proclaimed that we should “be worried . . . very worried” about global climate change (Kluger 2006). These discussions are now widespread across all media (from *Science* magazine to National Public Radio to the movie *An Inconvenient Truth*). Americans are fast becoming either worried sick or sick of worrying about global warming amid the endless predictions of the coming apocalypse [see, for example, the letters to the editor following *Time*’s story (Time Inc. 2006)]. They are also hearing a steady drumbeat of alarm about other forms of global environmental change, such as loss of biodiversity, species extinctions, and destruction of natural habitats. However, despite a broad scientific consensus that environmental degradation is caused by humans and will impact human health globally, very few exurb-dwelling, McMansion-living, large-lawn–watering, sport utility vehicle–driving, 100-mile-a-day–commuting, endangered-species consuming, therapeutic-shopping Americans acknowledge that their behaviors, and the policies allowing or even encouraging these behaviors, may be implicated and in need of change. Risk perception continues to focus on worries closer to home; a March 2006 Gallup survey (Gallup Poll 2006) reported that concern about global warming ranked lower than eight other environmental issues, such as pollution of rivers, lakes, and reservoirs and toxic waste.

How much should we worry about global climate change and other forms of environmental degradation? Any lingering doubts about its occurrence, as well as humankind’s influence, are quickly fading amid new reports of faster-than-expected glacial melting (Overpeck et al. 2006) and unprecedented rates of species loss, deforestation, desertification, and water shortages (Hughes et al. 2003; Jackson et al. 2001; Thomas et al. 2004). The much more challenging question is this: What steps can clinical practice and public health communities take now in an effort to address these challenges?

In this commentary, we argue that the evidence is inescapable that global environmental change is occurring and is caused by policies and human actions that are unsustainable. Global environmental change, in turn, has profound implications for human health. In a recent study, Patz et al. (2005) estimated that anthropogenic climate changes already claim at least 150,000 lives annually. At present, the health consequences of First-World excesses are being felt disproportionately by populations in the developing world. While politicians and business leaders delay, or search for painless solutions that require no sacrifice and have no impact on economic growth, clinicians and environmental health professionals must think rigorously about what can be done now. In this commentary, we summarize the evidence and issue a call to action. More specifically, we urge that *a*) changing current behaviors be the immediate priority while waiting for larger-scale policy and regulatory solutions; *b*) clinicians counsel their patients using tools that measure ecological footprints; *c*) professional organizations assist clinicians in developing and using such tools; *d*) carbon-and other environmental-labeling of products be implemented to facilitate behavior change; *e*) the environmental health community develop a global environmental health index for use in year-to-year monitoring that combines “planetary health” with human health; and *f* ) clinicians and environmental health professionals engage in the development and implementation of policy and regulatory solutions similar to those already proposed elsewhere (Brown 2006).

## A Brief Review of the Evidence for Global Environmental Change

Several terms, including “global warming,” “global climate change,” and more recently “global climate chaos,” have been used to describe the environmental consequences of collective human activity, most notably steady and historically large increases in greenhouse gases in the Earth’s atmosphere. We prefer to address a larger set of global environmental concerns, because other environmental changes that are global in scale are occurring; the consequences are not just about warming and not just about climate. Patterns of resource use (e.g., water, fossil fuels), habitat destruction (e.g., deforestation, desertification), and biodiversity and species loss (Millennium Ecosystem Assessment 2005), in addition to greenhouse gas levels, are closely interrelated, global in scale, human-caused, and have important implications for human health now and in the future. Several authors have noted the interconnectedness of apparently disparate trends: from our “addiction” to fossil fuels, to urbanization and suburban sprawl, war in the Middle East, terrorism, skyrocketing gas and oil prices, and global environmental change (Kunstler 2005; Wilson 2006). However, the public and many public health professionals continue to see these as separate issues rather than part and parcel of the same set of interrelated challenges.

A steady stream of opposing voices [for example, Lindzen (2006)] have argued that scientific uncertainties remain regarding the cause, extent, and consequences of climate change. However, the vast majority of scientists now agree that consensus has been achieved that global climate change is real and is caused by human behavior, and that these changes are moving faster than previously thought (Walther et al. 2002). Apparently, however, most Americans believe the dissenting voices; a recent *Time*/ABC News/Stanford University poll cited in the 3 April 2006 *Time* magazine shows that 64% of Americans thought that there was still “a lot of disagreement” about global warming (Kluger 2006). Undoubtedly, multimillion-dollar media campaigns by industries threatened by the implications of global warming have contributed to the mismatch between the public’s perception of doubt and the consensus among scientists (Doughton 2005; Gelbspan 2004). It is also likely that the journalistic norm of balanced reporting has contributed to the confusion; in a 2004 analysis of 340 articles in the U.S. prestige press on climate change, Boykoff and Boykoff (2004) found that 53% gave roughly equal attention to the humans-as-cause view and the “natural fluctuations” argument favored by industry.

Although scientific uncertainty remains about the extent, pace, and consequences of global climate change, there is no scientific uncertainty that fossil fuels are a finite, rapidly depleting, and increasingly expensive resource (Simmons 2005); deforestation and desertification are occurring rapidly throughout the world (Malhi and Phillips 2004); marine fisheries are declining globally (Caddy and Seijo 2005); species and biodiversity loss are occurring at an unprecedented rate, and this sixth global wave of extinction is the result of human activity (Leakey and Lewin 1995; Millennium Ecosystem Assessment 2005); current patterns of water use are unsustainable, with rapidly declining levels in the quantity and quality of surface and ground water stores (Gleick 2004); polar, Greenland, and Antarctic ice sheets are melting more rapidly than expected, resulting in sea levels rising more significantly and quickly than previously thought (Bindschadler 2006; Overpeck et al. 2006; Rignot and Kanagaratnam 2006; Velicogna and Wahr 2006); and levels of carbon dioxide in the atmosphere are increasing and at unprecedented levels (Yoganathan and Rom 2001). The debate about whether something must be done now should be effectively over.

## Global Environmental Change and Human Health

A detailed review of the evidence that global environmental change will affect human health is beyond our scope. Suffice it to say that the health consequences can be felt now and are not simply a problem for some future generation to tackle (Patz et al. 2005). These changes are a threat to human health.

The health impacts of global climate change include the direct effect of heat (e.g., heat stroke in the elderly); influences on severe weather, flooding, and drought; worsened cardiovascular and pulmonary mortality due to heat; the influence of heat on air pollution; differences in the distribution and effectiveness of vectorborne and waterborne disease transmission; threats to food production; and changes in the hydrologic cycle (McMichael et al. 2006; Patz et al. 2005). However, the health consequences of global environmental change are not limited to these. We have concerns for how current conceptions of disease causation may underestimate the true impact of “upstream” environmental changes that result in more indirect, complex, and potentially nonlinear processes. Glass and McAtee (2006) have written, concerning the 1998–2004 war in the Democratic Republic of the Congo:

This horrendous society-wide conflict is undoubtedly to blame for nearly 4 million deaths, but not because war ‘‘causes’’ death, but because war has fundamentally altered the social conditions of life in ways that create a new and lethal regime of risk.

We believe this thinking is also relevant to how global environmental change threatens the health and well-being of human populations. Many health impacts will result indirectly from social upheaval, environmental refugees fleeing a rising sea level, disruption of global food production and health care infrastructure, and conflicts over resources. Again to borrow from Glass and McAtee (2006), global environmental change will act as a risk regulator, fundamentally altering the environmental and social conditions of life in ways that create a new and lethal regime of risk for human populations around the world. Furthermore, as global environmental change contributes to ecosystem degradation, species loss, and possibly greater difficulties with food production (Houghton et al. 2001; White et al. 2001), these changes may lead to environmental scarcities, which in turn may increase violence and conflict (Homer-Dixon 1999), creating a cascading cycle of environmental change, scarcity, conflict, social disruption, and population morbidity and mortality.

A number of health impacts are highlighted here. In the summer of 2003 in Europe, 35,000 deaths were directly attributable to an historically unprecedented heat wave (Vandentorren et al. 2004). As ecosystems decline, food production will be threatened; for example, the Intergovernmental Panel on Climate Change (IPCC) estimates that crop yields could decline in some locations by 20–40%. In a world in which over a billion people already have inadequate nutrition, this number will undoubtedly grow, especially in view of the social disruptions that are likely to result. Such an argument is also directly applicable to concerns over water quantity and quality. Extreme weather events and changes in precipitation patterns will also contribute to the new and lethal regime of risk. Sea level rise (Hunt 2002; Senior et al. 2002) will cause us to change where we live and how we live.

The foregoing discussion highlights the need to distinguish individual health risks from population health risks in considering the impacts of global environmental change (Rose 1992). The magnitudes of the potential health impacts are much more readily apparent at the population level than at the individual level. The distribution of new and changing environmental risks in populations, the conditions of places, and the aggregate behaviors of human populations lead to fundamental changes in risk regimes that can threaten global gains in life expectancy and health.

## Human Behaviors Are the Cause of Global Environmental Change—Would Measurement Help?

All the global environmental changes that are occurring are caused by human behaviors, and these behaviors and their consequences differ dramatically among nations. For example, the United States, with 5% of the world’s population, produces 25% of the global carbon emissions that are fueling climate change. Whereas the average American uses 159 gallons of water per day, more than half of the world’s population lives on 25 gallons per person per day (Gleick 2004). China and India each have populations well over 1 billion. As those populations aspire to American-style patterns of consumption, the closed global ecology—already pushed to its limits in many places—could be degraded beyond repair. China has already surpassed U.S. levels of consumption in grain, meat, coal, and steel (Worldwatch Institute 2006).

There are several tools that can be used to help understand these impacts and lead the way to solutions. Ecologists have calculated the carrying capacity of the Earth with different lifestyle scenarios. Rees (1996) defines “carrying capacity” as the maximum number of organisms that an ecosystem can support and sustain without degrading the ability of that ecosystem to maintain that abundance in the future. Currently, humans are using 23% more resources than the planet can regenerate (Loh and Wackernagel 2004; Rees 1996). In economic terms, we are spending the Earth’s ecological principal, no longer living off the interest that can be regenerated every year. This is obviously not sustainable. Although no one would argue that the current global situation is ideal—with half of the population (3 billion people) living on ≤ $2/day (World Bank 2006) and close to 1 billion undernourished (Food and Agriculture Organization of the United Nations 2005)—how many Earths would it take to provide all the resources needed for the current global population to live like Americans? About five (Loh 2002). However, few policy makers are seriously discussing these issues. At a time when the U.S. market sees historically high gasoline prices, politicians offer $100 tax rebates rather than discouraging consumption and encouraging alternative fuels.

The ecological footprint is a tool to quantify the amount of water and land area necessary to sustain an individual or a population using available technology (Wackernagel et al. 2002). Earth has approximately 4.4 global acres (1 acre of biologically productive space, including both land and water, with world-average productivity) per person of biologically productive space. Currently, each of 6.2 billion people uses an average of 5.4 global acres, yielding a deficit of 1 global acre per person. Earth’s resources are not distributed evenly, however. Bangladeshis, on average, use only 1.2 global acres per person, whereas Americans use 24 global acres. E.O. Wilson recommends that we reserve roughly half of the available global acres for the world’s wildlife and nonhuman living organisms to maintain biodiversity (Wilson 1993, 2002), leaving 2–2.5 global acres per person. In addition to its utility in guiding individual behavioral choices, the ecological footprint also can be used by cities or nations, for example, to explore different options and learn which of their own human activities have the greatest impacts on sustainability—and which are most critical to change as soon as possible [for one way of calculating ecological footprints, see Redefining Progress (2006)].

## Clinical Practice and Global Environmental Change

What do clinical practitioners do when the condition of places, often at long distances, becomes a threat to the health of their patients? What do clinicians do when average behaviors in populations—not the behavior of my own patient—pose significant risks to the community? Traditionally, the answer has been that these are not issues where clinicians have a defined role; rather, development of solutions is left to policy makers and the private sector. We believe it is time for clinicians to become engaged, to “think globally and act locally” (Dubos 1968). This requires clinicians to see the interconnectedness between our own behavioral choices and those of our patients and the health risks that occur in aggregation and over long distances within the context of a finite and closed ecosystem. Any given individual might change his or her own behavior for the better, but could still be at high risk for the health impacts of global environmental change because of the average condition of other places and behaviors of distant populations. For example, behaviors in industrialized countries are threatening whole communities on low-lying Pacific islands due to sea level rise (Gaffin 1997), and the conditions of certain marine ecosystems (e.g., coral reefs) are threatening marine fisheries at great distances (Myers and Worm 2003; Walther et al. 2005; Worm et al. 2005).

The NIH (National Institutes of Health) Roadmap has a clear focus on patients and the translation of research findings from laboratory to clinic (Zerhouni 2003). Although population health is mentioned in the roadmap, for example, at the National Institute of Environmental Health Sciences (Schwartz 2005a, 2005b), the emphasis is on bringing interventions to improve health to individual patients. If we agree that global environmental change is occurring and that it is a profound threat to human health, we urge a matching focus on population health issues. If we agree that the aforementioned threats are real, then we cannot rely solely on individual-level interventions. The much anticipated advances in molecular and genetic medicine will do the human race no good if the planet becomes unable to sustain human life.

Clinicians, even those trained in disciplines not focused on preventive medicine, have long practiced prevention. We ask patients to stop using tobacco, to exercise, and to eat healthy diets, even if they may be currently asymptomatic and healthy. Clinical practice involves issuing recommendations to our patients today to prevent future individual health risk. How about when my behavior poses a threat not to me but to others? There are preventive practices that set a precedent for this as well. For example, when I ask my patient if he owns a handgun and if it is secured properly in the home, I am trying to minimize the risk that the handgun poses to family members (Bukstein et al. 1993) and the larger community, not only to my patient.

Many behaviors in the United States and other industrialized countries are now posing health risks to human populations around the world. We believe that clinicians should counsel their patients and communities on behavior change issues and use the ecological footprint method as a guiding tool. We are not suggesting that all clinicians become experts in climatology or behavior change; however, clinicians are already on the front lines of prevention with respect to many behavioral issues. They have experience in behavior change and counseling, and they have credible voices on health-related issues (as opposed to politicians or industry, for example). Clinicians have used health risk assessments to evaluate sets of individual risk factors of relevance to individual and family health. As guided by analysis of ecological footprints, clinicians could counsel their patients about unsustainable behaviors such as the need to consume food and other materials more locally, eat lower on the food chain, reduce carbon emissions in transportation and in the home, work and recreate closer to home, live in homes with lower square footages, and advocate residential models designed for optimal sustainable development such as the new urbanism [see, for example, NewUrbanism.org (2006)]. Also, Donohoe (2003) suggested a number of actions for health professionals to combat environmental degradation and social injustice.

To assist clinicians in these efforts, we recommend two additional actions. First, professional organizations (e.g., American Medical Association, American College of Physicians, American Public Health Association, American Academy of Physician Assistants and Nurse Practitioners) should become involved by advocating that their members start living sustainably themselves, counsel their patients on sustainable behaviors, and begin committees for development and implementation of clinical tools. Second, to aid in behavior change, we believe that products should be labeled for their carbon emissions in both production and in use, as well as other environmental impacts, in an effort to internalize environmental costs. Although there are considerable methodologic challenges for this recommendation, such labeling would greatly assist consumers in making responsible choices. This recommendation would also require policy and regulatory action.

There are precedents for clinicians engaging in large threats much outside the scope of clinical practice. In 1962, the world saw in the Cuban missile crisis the hair-trigger nature of nuclear war, and civil defense plans were well under way to prepare communities for the threat of a nuclear attack. However, members of the medical community were concerned about the illusion of ever being medically “prepared” for the catastrophe of a nuclear war and the critical need to sound an alarm for the wider public. Articles appeared and lectures and meetings began to occur (Ervin et al. 1962; Sidel et al. 1962) that culminated in 135,000 clinicians organizing into International Physicians for the Prevention of Nuclear War. By publishing a number of papers extolling the grim human and ecological costs of even a limited nuclear war that continued into the 1980s (Chalmers et al. 1984; Chivian et al. 1988; Litwin 1985; Lown and Chazov 1985; Lown et al. 1981; Smith 1984), member groups played a major role in shifting the policy debate and public attitudes on nuclear war, resulting in the 1985 Nobel Peace Prize (Lown et al. 1981), and continued work on related topics (Hu et al. 1992; Loretz 2004). In this context, global climate change can be seen as nuclear war “in slow motion”—potentially globally devastating with enormous consequences for human health and deserving of the attention and action of the public health and health care communities. Because it is outside the comfort zone of many practitioners to be engaged in social issues outside the office, it is even more important that we debate the ways to transform our definitions of clinical practice now. Just as the sanitary movement of the 19th century required health practitioners to come out of the hospitals and engage with the larger world to stop tuberculosis, cholera, and pellagra, we argue that similar efforts are now required to address global environmental change.

## Roles for Environmental Health Practitioners

We believe the public health community must learn from the successes of economists and develop new metrics that can facilitate public health goals. Politicians and policy makers eagerly await reports of the latest indices of economic performance, because economists have defined and developed the metrics of national and global economic output and success. The public health community should develop their own metrics for conveying the state of the environment and population health both globally and regionally. A carefully designed global environmental health index could be developed and adjudicated by a panel of independent scientists and public health experts. It would be designed to measure the health of a world we all would want to live in—in which our lifestyles can be sustained—and to measure the impact of “planetary health” on human health. This metric could be used to track performance, to compare the success of national efforts, and to motivate nations to focus on the health of our global environment and its people rather than our economic output.

There are already examples of similar metrics such as the Living Planet Index for species loss and national and world ecological footprints (Loh and Wackernagel 2004). The world ecological footprint, for example, is calculated in global hectares per person and is a comprehensive metric that includes population; a food, fiber, and timber footprint; an energy footprint (including carbon emissions, fuel wood, nuclear, and hydro power); built-up land; total biocapacity; and ecological deficits (including water withdrawals and resources) (Loh and Wackernagel 2004). These metrics could be modified to better link the impact of planetary health to human health, with the input of public health professionals, clinicians, ecologists, economists, and policy makers. A suitable global environmental health index would offer a policy-relevant tool to evaluate interventions designed to improve the human health impact of environments. Environmental health practitioners are ideally suited to play key roles in the development and implementation of these new metrics, as well as policies and regulations to protect the global environment and reduce unsustainable behaviors.

## Policy and Regulatory Approaches

We do not want to overstate the impacts that we expect from individual-level behavior change. As explained by Glass and McAtee (2006), “while distal social conditions are more difficult to observe, they are ultimately more important in determining disease rates in populations.” They conclude that distal social conditions, such as control and manipulation of laws, norms, rules, and life conditions, may have greater impact on the public’s health than the control of proximate causes. Thus, it is essential that policy and regulatory solutions be developed, because we cannot simply rely on voluntary behavior changes from individuals. Public problems require public solutions.

Global environmental changes are being caused by two main factors—population growth and unsustainable consumer behaviors—and policies allow and encourage the choices that influence or make possible individual behaviors. We have been influenced by the detailed, comprehensive solutions of Lester Brown, most recently in his book *Plan B 2.0* (Brown 2006). As he sees it, the two most critical challenges are to restructure taxes and reorder fiscal priorities to provide for basic social and earth restoration goals. Brown (2006) argues that to achieve our earth restoration goals we must first meet a set of basic social goals that includes universal primary education, adult literacy, school lunch programs in the 44 poorest countries, assistance to preschool children and pregnant women in these countries, reproductive health and family planning, universal basic health care, and provision of condoms. These social goals will stabilize population growth, prevent the rapidly accruing numbers of failed states, and dramatically reduce poverty, all critical to stopping the ongoing global environmental changes. At the same time, earth restoration goals include proposals for reforestation, protecting topsoil on cropland, restoring rangelands, stabilizing water tables, restoring fisheries, and protecting biological diversity. He estimates that the social and earth restoration goals can be achieved for $161 billion annually and presents arguments for why this sum is achievable. We urge our colleagues to give this book to their spouses and partners, children, friends, colleagues, co-workers, and patients, and to discuss and debate its proposals.

A detailed discussion of adaptation and mitigation as defined by the IPCC is beyond our scope. In brief, adaptation/response options in general could be categorized as share the loss, bear the loss, modify the events, prevent the effects (structural/technologic, legislative/regulatory/financial, institutional/administrative, market based, on-site operations), change use, change location, research, and education and behavioral (White et al. 2001). Mitigation includes interventions to reduce greenhouse gas emissions while considering sustainable development and equity issues (Banuri et al. 2001), as well as carbon dioxide capture and storage (Metz et al. 2005) and other options. Although clinicians and public health professionals not directly engaged in response to global environmental change can participate on scientific panels and use their voting rights to advocate for specific adaptation and mitigation options, it is likely that the influence they have with individual patients and communities may have the greatest potential impact.

Unfortunately, even if we were to stop putting greenhouse gases into the atmosphere today, there is already sufficient momentum in the climate system to ensure that significant continued warming and climate change would occur. We realize some adaptation strategies are probably necessary. Without significant reductions in greenhouse gas emissions, however, no amount of adaptation will adequately protect populations from sizeable harm. We also question the morality of primarily focusing on adaptation to climate change because it presumes that those with resources will be able to pay for adaptation/protection strategies while continuing the unsustainable behaviors that condemn those without resources to suffer the consequences.

To assist clinicians with behavior change, we should develop state, federal, and local government policies to incentivize behaviors that lead to sustainable and responsible environmental impacts. This could include carbon-and other environmental-labeling of products, allowing consumers to make purchasing decisions with information of similar utility to that required for the nutritional content of food by the Food and Drug Administration. It is unlikely that industry will adopt this strategy voluntarily without regulatory mandates. Policy makers should also consider tiered pricing for electricity and other environmentally damaging activities; this would also have the benefit of mitigating the impact of environmental protections on the poor. In tiered pricing, electricity is cheapest for the first kilowatts consumed in order to meet basic needs, and progressively higher as consumption increases. In this way, costs could be decreased for the poor and used to inhibit consumption in the higher users (Hancock 2006). Another innovative strategy would be to implement a “cap and trade” scheme for individuals to reduce energy use and consequent greenhouse gas emissions. Under this plan, all individuals would be issued a “carbon account” starting with an equal amount of carbon units deemed necessary to meet basic needs. Carbon units would be deducted from the account for every purchase of gasoline or heating fuel, for example. Individuals choosing to drive cars requiring more gasoline or heat large houses would need to purchase additional carbon credits. Individuals who conserve fuel would have excess credits that they could then sell back to the system for a profit (Starkey and Anderson 2005).

We acknowledge the challenges faced by politicians and policy makers when faced with the need for fundamental changes in lifestyles. It is not easy to ask constituents to give up individual freedoms and luxuries. However, many analysts have argued that technologic solutions to many of these problems are unlikely (Kunstler 2005); we must change the way we live. A hydrogen-, ethanol-, or wind-based economy is not likely to be the sole solution, but there is certainly opportunity for research into these areas to make them part of a solution package that involves both lifestyle changes and improved technology. Some have advocated nuclear power as a potential solution. We are concerned about substituting one set of environmental hazards for another. Further, the rush to nuclear power has the added disadvantage of giving the impression that technology can provide the magic bullet to obviate the need for changing the way we live. Finally, nuclear power may decrease carbon emissions but will not prevent ecosystem destruction and species loss.

Considerable controversy has arisen about the effectiveness of market-based solutions to ecological problems. If the actual costs of environmental impact can be calculated and factored into products, then market-based strategies may have value. Most often, market-based incentives have failed to fully value environmental, ecological, and related health impacts (Kimbrell 1998). The result is a massive ecological debt, not accounted for in current pricing and policies, to pay for our current lifestyles. In short, market-based strategies may play a role, but not if markets fail to take account of the true economic costs of environmentally destructive practices. Moreover, we cannot wait for a market signal such as high energy prices to motivate mass behavior change. If we do, the environmental impacts may be too large and the action too late. Instead of investing hundreds of billions of dollars gaining access to and protecting dwindling oil supplies—only ensuring perhaps a few more decades of increasingly expensive oil—we should be investing in wind, solar, and other renewable sources of energy, in terms of both research and infrastructure.

## Conclusions

We believe the challenges we face are unprecedented in human history. We also believe that clinicians and the environmental health community have a professional obligation to become advocates for change at the individual and policy levels. We have made a number of proposals and would welcome a debate on how environmental health professionals and all clinicians should proceed in engaging these issues. The work of Dubos [e.g., Dubos (1968)] and others has given us cause to be optimistic. Humans are capable of social evolution, which allows us to rethink our actions and make relatively rapid changes that could help to lead to a stabilization of the global environment and human health risks. In the 1960s, America achieved a landing on the moon thanks to a concerted national commitment with timely leadership. We are once again called upon to rise to that level, but this time, rather than merely leaving our planet, we must try to save it.
